# Supplementary material for: Modeling glioblastoma heterogeneity as a dynamic network of cell states
Source: Mol Syst Biol. 2021 Sep 16;17(9):e10105. doi: 10.15252/msb.202010105 (PMC8444284; doi:10.15252/msb.202010105)
Supplement: Supplementary file 6 — Source Data for Figure 5 [file MSB-17-e10105-s004.zip › Figure5A_sourcedata/GSEA_3071/nosigdb_stateI.GseaPreranked.1622795145664/NB_KI_CLUSTER11.html]

Details for gene set NB\_KI\_CLUSTER11[GSEA]

|  || Dataset | state13071 |
| Phenotype | NoPhenotypeAvailable |
| Upregulated in class | na\_pos |
| GeneSet | NB\_KI\_CLUSTER11 |
| Enrichment Score (ES) | 0.52384007 |
| Normalized Enrichment Score (NES) | 2.5398488 |
| Nominal p-value | 0.0 |
| FDR q-value | 0.0 |
| FWER p-Value | 0.0 |
Table: GSEA Results Summary

  

Fig 1: Enrichment plot: NB\_KI\_CLUSTER11      
 Profile of the Running ES Score & Positions of GeneSet Members on the Rank Ordered List

  

| PROBE | GENE SYMBOL | GENE\_TITLE | RANK IN GENE LIST | RANK METRIC SCORE | RUNNING ES | CORE ENRICHMENT || 1 | RARRES3 |  |  | 22 | 1.575 | 0.0612 | Yes |
| 2 | S100A4 |  |  | 30 | 1.506 | 0.1237 | Yes |
| 3 | SLC2A3 |  |  | 109 | 1.060 | 0.1467 | Yes |
| 4 | CIB1 |  |  | 128 | 0.965 | 0.1829 | Yes |
| 5 | TGFB1 |  |  | 165 | 0.884 | 0.2104 | Yes |
| 6 | S100A6 |  |  | 204 | 0.815 | 0.2344 | Yes |
| 7 | B2M |  |  | 241 | 0.744 | 0.2560 | Yes |
| 8 | EMP3 |  |  | 260 | 0.726 | 0.2819 | Yes |
| 9 | S100A11 |  |  | 275 | 0.707 | 0.3081 | Yes |
| 10 | IRF1 |  |  | 276 | 0.705 | 0.3384 | Yes |
| 11 | AC020916.1 |  |  | 303 | 0.669 | 0.3596 | Yes |
| 12 | ISG15 |  |  | 328 | 0.647 | 0.3804 | Yes |
| 13 | FLNA |  |  | 373 | 0.614 | 0.3941 | Yes |
| 14 | AKAP13 |  |  | 375 | 0.611 | 0.4200 | Yes |
| 15 | KLF2 |  |  | 428 | 0.571 | 0.4295 | Yes |
| 16 | PSMB9 |  |  | 437 | 0.560 | 0.4512 | Yes |
| 17 | SP100 |  |  | 466 | 0.538 | 0.4662 | Yes |
| 18 | CASP4 |  |  | 585 | 0.460 | 0.4519 | Yes |
| 19 | THBS1 |  |  | 613 | 0.446 | 0.4632 | Yes |
| 20 | S100A10 |  |  | 673 | 0.422 | 0.4643 | Yes |
| 21 | BST2 |  |  | 677 | 0.421 | 0.4816 | Yes |
| 22 | ARPC1B |  |  | 743 | 0.400 | 0.4800 | Yes |
| 23 | PNRC1 |  |  | 769 | 0.392 | 0.4896 | Yes |
| 24 | TSPO |  |  | 826 | 0.373 | 0.4895 | Yes |
| 25 | IFITM2 |  |  | 836 | 0.370 | 0.5027 | Yes |
| 26 | FOSB |  |  | 854 | 0.364 | 0.5134 | Yes |
| 27 | ZC3H12A |  |  | 872 | 0.358 | 0.5238 | Yes |
| 28 | MYL12A |  |  | 1053 | 0.316 | 0.4856 | No |
| 29 | IFI16 |  |  | 1062 | 0.315 | 0.4967 | No |
| 30 | GLIPR1 |  |  | 1346 | 0.260 | 0.4264 | No |
| 31 | MSN |  |  | 1379 | 0.255 | 0.4281 | No |
| 32 | EEF1D |  |  | 1769 | -0.290 | 0.3285 | No |
| 33 | TGIF1 |  |  | 1955 | -0.313 | 0.2886 | No |
| 34 | CTSC |  |  | 1975 | -0.315 | 0.2967 | No |
| 35 | FOSL2 |  |  | 2015 | -0.319 | 0.2991 | No |
| 36 | PFN1 |  |  | 2305 | -0.373 | 0.2318 | No |
| 37 | IER2 |  |  | 2587 | -0.436 | 0.1696 | No |
| 38 | ARID5B |  |  | 2826 | -0.511 | 0.1229 | No |
| 39 | LITAF |  |  | 3247 | -0.843 | 0.0381 | No |
| 40 | GYPC |  |  | 3267 | -0.877 | 0.0703 | No |
Table: GSEA details [plain text format]

  

Fig 2: NB\_KI\_CLUSTER11: Random ES distribution      
 Gene set null distribution of ES for **NB\_KI\_CLUSTER11**
